# Attenuation of postprandial blood glucose in humans consuming isomaltodextrin: carbohydrate loading studies

**DOI:** 10.1080/16546628.2017.1325306

**Published:** 2017-05-24

**Authors:** Tsuyoshi Sadakiyo, Yuki Ishida, Shin-ichiro Inoue, Yoshifumi Taniguchi, Takeo Sakurai, Ryodai Takagaki, Mayumi Kurose, Tetsuya Mori, Akiko Yasuda-Yamashita, Hitoshi Mitsuzumi, Michio Kubota, Hikaru Watanabe, Shigeharu Fukuda

**Affiliations:** ^a^ R&D Center, Hayashibara Co., Ltd., Okayama, Japan

**Keywords:** Isomaltodextrin, soluble dietary fiber, carbohydrate loading, blood glucose, everted rat intestinal sac

## Abstract

**Background**: Isomaltodextrin (IMD) is a novel highly branched α-glucan and its function as a soluble dietary fiber is expected.

**Objective**: The goal of this study was to evaluate the effects of IMD on postprandial glucose excursions in healthy people and to make the mechanism clear.

**Design**: Twenty-nine subjects ingested a solution containing maltodextrin (MD) or sucrose with or without IMD. Fourteen subjects ingested a solution containing glucose with or without IMD. Blood glucose concentrations were then compared between the groups. Furthermore, *in vitro* digestion, inhibition of digestive enzymes, and glucose absorption tests were conducted.

**Results**: IMD attenuated blood glucose elevation in the subjects with blood glucose excursions at the high end of normal following the ingestion of MD or sucrose or glucose alone. This effect of 5 g IMD was most clear. IMD was digested partially only by small intestinal mucosal enzymes, and maltase and isomaltase activities were weakly inhibited. Furthermore, IMD inhibited the transport of glucose from mucosal side to serosal side.

**Conclusions**: IMD attenuated postprandial blood glucose, after the ingestion of MD or sucrose or glucose. As one of the mechanism, it was suggested that IMD inhibited the absorption of glucose on small intestinal mucosal membrane.

## Background

**Figure 1.**
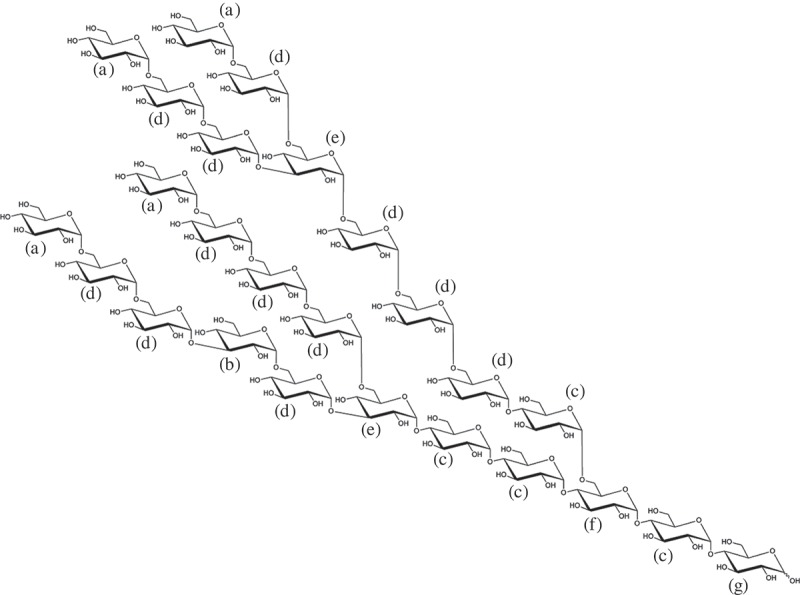
The putative structure of IMD. (a) Nonreducing end α-Glcp; (b) 1,3-linked α-Glcp; (c) 1,4-linked α-Glcp; (d) 1,6-linked α-Glcp; (e) 1,3,6-linked α-Glcp; (f) 1,4,6-linked α-Glcp; (g) reducing-end Glcp.

Isomaltodextrin (IMD) is a novel highly branched α-glucan produced enzymatically from starch using α-glucosyltransferase and α-amylase derived from *Paenibacillus alginolyticus* PP710 [1]. IMD consists only of glucose units with approximately 17% α-1 glucosidic linkages (nonreducing end group), 3% α-1,3 glucosidic linkages, 19% α-1,4 glucosidic linkages, 49% α-1,6 glucosidic linkages, 7% α-1,3,6 glucosidic linkages, and 5% α-1,4,6 glucosidic linkages, and the average molecular weight was approximately 5000 (Figure 1). IMD is a white powder with no coloration and odor; it is freely soluble in water and is unlikely to alter the taste or flavor. The dietary fiber content of IMD measured using the enzyme-HPLC method (AOAC 2001.03) is more than 80% as dry solid basis [2]. Resistant maltodextrin (RMD) and polydextrose are also recognized as glucose-based soluble dietary fibers, but IMD is different in that it consists of only α glucosidic linkage and has many α-1,6 glucosidic linkages compared with these materials. Therefore, IMD is expected to exhibit different physiological effects.

The development of excessive postprandial hyperglycemia is a risk factor for type 2 diabetes mellitus [3]. According to a report by the International Diabetes Federation, the number of people aged 20–79 years with diabetes was approximately 7.2 million in Japan in 2013 and about 380 million people worldwide [4]. In most developed countries, poorly managed diabetes mellitus is associated with complications such as diabetic neuropathy, renal failure, blindness, macrovascular disease, and mortality [3]. It is therefore quite important to manage excessive postprandial glucose excursions during daily life. With the functional properties of a soluble dietary fiber, IMD is expected to attenuate postprandial blood glucose concentrations, as occurs with several other dietary fibers [5,6].

The present study was undertaken to evaluate the postprandial blood glucose lowering effects of IMD when it is consumed with various carbohydrates in healthy humans. First, to investigate the effect of IMD administered together with maltodextrin (MD) or sucrose on postprandial glucose, loading studies were performed. Then the glucose loading study was planned to investigate effect of the IMD lower dose. Furthermore, to analyze the mechanism of postprandial glucose attenuation, *in vitro* digestion, inhibition of digestive enzymes, and glucose absorption tests were conducted.

## Materials and methods

### Test substance and loaded carbohydrates

The test substance was IMD (Hayashibara Co, Ltd., Okayama, Japan). The dietary fiber content of the IMD sample measured using enzyme-HPLC method (AOAC 2001.03) was 84.1% as dry solid basis. The loaded carbohydrates were MD (Pinedex #1, Matsutani Chemical Industry Co., Ltd., Hyogo, Japan), sucrose (Caster Sugar, Dai-Nippon Meiji Sugar Co., Ltd., Tokyo, Japan), and glucose (Fuji Cristar, Kato Kagaku Co., Ltd., Aichi, Japan). The quantities of test substance/carbohydrates were used as dry solid basis in this study. During the human study, each subject ingested a solution containing the test substance/carbohydrates. The solution volumes were 200 mL in MD and glucose loading study, and 300 mL in sucrose loading study. Because IMD was almost free of coloration, taste, and odor, and the quantity added did not exceed 5% of the solution, it was judged improbable that subjects would be able to distinguish its presence/absence in the solutions used.

### Subjects of MD and sucrose loading study

Loaded carbohydrate doses were 46.8 g of MD and 100 g of sucrose. Added IMD were 9.6 g. Subjects included 30 healthy male and female employees of Hayashibara Co., Ltd. who agreed to participate in the study and met the study entry criteria. Key inclusion criteria included no history of abnormal glucose metabolism and no ongoing use of drugs that affect blood glucose. Subjects who met the requirements were judged healthy and eligible by a doctor and were enrolled in the study.

In the MD loading study, one subject had an HOMA-R (an index of insulin resistance) of 3.5 after ingestion of the first carbohydrate solution. This subject was withdrawn from all subsequent studies. After completion of the sucrose loading studies, one subject was found to have taken a drug that can compromise insulin activity immediately before the sucrose loading study, and this data was omitted from the sucrose loading analysis. Analysis was conducted on data from 29 subjects in the MD loading study and from 28 subjects in the sucrose loading study. Characteristics of subjects in each test in each study are summarized in Table 1.

**Table 1.** Characteristics of subjects in each test.

		MD loading study		Sucrose loading study		Glucose loading study	
		All	≥70 mg/dL^a^	All	≥75 mg/dL^a^	5 g IMD	10 g IMD
Number of subjects	Total	29	9	28	7	13	14
	Male	24	8	23	6	10	11
	Female	5	1	5	1	3	3
Age (y)		40.1 ± 6.4	42.0 ± 5.7	40.4 ± 6.4	44.3 ± 6.9	41.7 ± 6.7	40.8 ± 7.3
BMI (kg/m^2^)		21.9 ± 2.1	21.1 ± 2.7	21.9 ± 2.1	20.2 ± 2.7	22.9 ± 2.4	22.4 ± 2.9
Mean peak level of blood glucose (mg/dL)	Measured level	140.6 ± 25.0	171.8 ± 10.4	144.6 ± 22.1	171.7 ± 15.8	190.6 ± 11.9	190.1 ± 11.7
	Δ	54.2 ± 23.1	82.4 ± 9.6	59.3 ± 20.3	84.7 ± 7.7	95.0 ± 8.5	94.7 ± 8.2

Values are shown as mean ± SD.

Δ is expressed as the value that subtracted fasting level from random level.

^a^Is data of subject that peak Δ(blood glucose) showed ≥70 mg/dL in MD loading study or ≥75 mg/dL in sucrose loading study.

### Subjects of glucose loading study

Loaded glucose dose was 50 g. Added IMD was 5 or 10 g. Subjects were recruited as well as MD and sucrose loading study, and 29 people who could participate were finally chosen. Fifteen of the 29 subjects with the highest change from fasting to peak blood glucose in ingestion of glucose alone were selected for ingestion of glucose with IMD. Exclusion criteria for the glucose loading study were in place to enable selection of healthy subjects likely to show a blood glucose increase within a certain range whilst also taking their safety into consideration. Acceptable blood glucose values commensurate with the existing guidelines [3,7,8] for hypoglycemia during hunger as well as fasting and hyperglycemia during hunger as well as fasting, random and postprandial tests, were the basis of our selection criteria in this study. However, it is necessary to revise in capillary blood glucose value used in this test because these guidelines make the venous blood glucose value as the standard. Modifications were made to guideline random and postprandial (120 min after ingestion) blood glucose ranges in accordance with a correction factor reported by Larsson-Cohn (multiplied by 1.35) [9] to account for an overestimation that is characteristic when testing for glucose in capillary blood compared with venous blood (as occurred in this study). Results from the glucose loading study excluded data from subjects with the following blood glucose results: (a) fasting <60 mg/dL or ≥126 mg/dL; (b) 120 min postprandial ≥189 mg/dL; and (c) random ≥270 mg/dL.

Based on the data derived from ingesting glucose alone, 15 subjects were selected for the glucose loading study. These subjects ingested glucose + IMD (10 g and later 5 g of IMD). One subject with a blood glucose of 143 mg/dL before glucose + IMD (10 g) ingestion became ineligible to continue the study based on the prespecified exclusion criterion (≥126 mg/dL). Another subject with an acceptable glucose + IMD (10 g) postprandial blood glucose response had a 120 min postprandial blood glucose of 192 mg/dL after ingestion of glucose + IMD (5 g) which exceeded the exclusion criteria (≥189 mg/dL). However, this subject’s hyperglycemia was considered to reflect an influence on their physical condition limited to the day concerned; therefore, only the abnormal data for ingestion of glucose + IMD (5 g) were excluded from analysis. Characteristics of subjects in this test in each study are summarized in Table 1.

### Design of each carbohydrate loading study

The MD loading study was performed first followed by the sucrose loading study. If the IMD attenuated the post ingestion blood glucose increase, the glucose loading study was to be performed to investigate the mechanism of action and effect of the IMD lower dose.

MD and sucrose loading study: The MD and sucrose loading studies were performed in a single-blind crossover fashion and subjects were blinded and randomly allocated to each group. Information about ingested substances was controlled strictly by the examiner, without informing to the subjects by the end of the study. Each subject was instructed to begin fasting by 9:00 pm the day before the study and to consume no drinks (only small amounts of water if necessary for health reasons) until the solution ingestion of next morning. A blood sample was drawn from each subject by 9:00 am on the test day and the carbohydrate solution was ingested within a few minutes. Blood sampling was performed from the median cubital vein at 15, 30, 45, 60, 90, and 120 min after solution ingestion. The series of steps for each of the solutions were conducted with intervals of more than 1 week.

Glucose loading study: In both the MD and sucrose loading studies, post ingestion blood glucose attenuation effects of IMD were of a greater magnitude in the subjects who exhibited a more pronounced blood glucose increase, and subjects with same phenomenon were selected for the glucose loading study in the following way. After the fasting period, blood samples were drawn and the solution of glucose was ingested similarly to the MD or sucrose loading studies. Blood samples were collected from a fingertip capillary at 30, 45, 60, 90, and 120 min after ingestion. On the basis of the peak of change in blood glucose (Δ(blood glucose)) recorded at those times, the 15 subjects with the highest peak Δ(blood glucose) were selected for the study. These 15 subjects then ingested about a glucose solution containing 10 g IMD and repeated the same sequence of steps. After an interval of more than one week, the same test procedure was performed using a glucose solution containing 5 g IMD.

### Measurements of each carbohydrate loading study

Analysis of blood samples collected during the MD and sucrose loading studies were assigned to the Okayama Medical Association, Medical Center (Okayama, Japan). Blood glucose was measured as plasma glucose concentration using the glucose oxidase (GOD) method. Serum insulin was measured by enzyme immunoassay. In the glucose loading study, a lancing device (Safe-T-Pro Uno, Roche Diagnostics, Basel, Switzerland) was used for the self-sampling of capillary blood, and blood glucose concentration was self-measured with a meter (ACCU-CHEK Aviva Nano, Roche Diagnostics, Basel, Switzerland). The conductor attended in the self-measurement to check the condition of subjects, to confirm of the processes, to record measured values.

### In vitro IMD digestion test

To estimate the digestibility of IMD in humans, digestion tests *in vitro* were carried out by using salivary amylase, synthetic gastric juice, pancreatic juice amylase, and rat small intestine mucosal enzyme. Human saliva was supplied by the examiner. Synthetic gastric juice was prepared by the method of Fujita et al. [10]. Porcine-derived pancreatic α-amylase (Roche Diagnostics, Basel, Switzerland) was purchased. Rat intestinal acetone powder (Sigma-Aldrich, Tokyo, Japan) was used as a small intestine mucosal enzyme. The *in vitro* digestion studies except the small intestine mucosal enzyme were performed by the method of Fujita et al. [10]. The digestion test of small intestine mucosal enzyme was carried out by the method of Ichikawa et al. [11] and Oku et al. [12]. The controlled carbohydrate was used above-mentioned MD in this test.

### Inhibition test of digestive enzymes

Maltose (MALTOSE HHH, Hayashibara Co, Ltd., Okayama, Japan), isomaltose (reagent grade, Hayashibara Co, Ltd., Okayama, Japan), or sucrose (special grade reagent, Wako Pure Chemical Industries, Ltd., Osaka, Japan) were used as substrates. Rat intestinal acetone powder (Sigma-Aldrich, Tokyo, Japan) was used as an enzyme source.

Rat intestinal acetone powder was added to 50 mM sodium maleate buffer (pH 6.6). After centrifugation, supernatant fraction was collected as enzyme extract. The supernatant fraction was diluted to be 0.045 or 0.09 u/mL as activity of maltase and isomaltase and 0.09 or 0.18 u/mL as activity of sucrase. One unit of maltase or isomaltase in this study was defined as the quantity of the enzyme needed for formation of the reduced sugar equivalent to 2 μmol glucose from substrate (maltose/isomaltose) under conditions of 37°C for 1 min. One unit of sucrase in this study was defined as the quantity of the enzyme needed for formation of the reduced sugar equivalent to 1 μmol glucose from sucrose under same conditions.

This study was conducted by the method of Dahlqvist [13]. Two substrate concentrations (90 or 180 mg/dL) were set in considering the change of carbohydrate level in jejunum. The solutions containing of substrate and 0 or 60 mg/dL of IMD in 50 mM sodium maleate buffer (pH 6.6) were made. When the substrate concentration was 90 mg/dL, maltase or isomaltase activity was set to 0.045 u/mL and sucrase activity was set to 0.09 u/mL. When the substrate concentration was 180 mg/dL, maltase or isomaltase activity was set to 0.09 u/mL and sucrase activity was set to 0.18 u/mL. Two mL of the solution were added 0.2 mL of enzyme extract, incubated at 37°C for 30 min. Samplings (1.0 mL) were conducted before and after reaction, then the reaction was terminated at 100°C and 10 min. Glucose concentrations of each sample were measured with GOD method after they were diluted to appropriate concentrations.

The inhibitory ratio of IMD (%) was calculated using the following formula: (*A* + *B* − *C*)/(*A* + *B*) × 100, where *A* is the glucose production of the enzyme with a substrate, *B* is the glucose production of the enzyme with IMD, and *C* is the glucose production of the enzyme with a substrate and IMD. If the calculated value was negative, we judged that inhibition of IMD did not occur.

### Glucose absorption test using everted rat intestinal sac

The experimental equipment was made referring to the method reported by Tsuchiya et al. [14]. Twelve male Wistar rats weighing about 250 g (supplied by CLEA Japan, Inc.) were used. Rats were sacrificed under pentobarbital anesthesia (intraperitoneal administration of 60 mg/kg-body weight) after fasting for four hours. Then the intestine was excised quickly. Segment of the intestine used in this study was about 8 cm length from 16 cm point to the ileum side from Treitz ligament referring to the previous studies [15,16]. The segments were rinsed with cool Krebs–Ringer bicarbonate buffer solution (KRB buffer solution: 118 mM NaCl, 4.7 mM KCl, 2.5 mM CaCl_2_, 1.2 mM KH_2_PO4 1.2 mM MgSO_4_, 25 mM NaHCO_3_, pH 7.4) bubbled with 95% O_2_/5% CO_2_. The segments were everted, and inserted a polypropylene tube at proximal side end. Then the segments were ligated on 1 cm point from proximal side end. Distal side of the segments were ligated to make 5 cm length of intestine sacs, and filled with 0.5 mL of KRB buffer solution. The sacs were placed in 20 mL of KRB buffer solution containing 10 mM glucose. The incubation bubbled with 95% O_2_/5% CO_2_ was carried out at 37°C for 2 h. KRB buffer solution around sacs with/without IMD (60 mg/dL) was used in this study (each *n* = 6). Sampling (5 μL) of the serosal side solution in sacs were conducted at 0, 30, 60, 90, 120 min after incubation. Glucose C II Test Wako (Wako Pure Chemical Industries, Ltd., Japan) was used in measurement of glucose concentration of samples.

### Ethics

This study was conducted in compliance with the Declaration of Helsinki, and approved at the Ethics Committee of Hayashibara Co., Ltd. (approval number 126,168). All study subjects were given full information about the importance, purpose and experimental protocol, and provided written informed consent about entry and publishing for each study.

The animal study was approved by the Animal Care and Use Committee of the R&D Center of Hayashibara Co., Ltd. (approval number hb1508-02, hb1511-01) and conducted in compliance with in-house Regulations on Animal Experimentation at the R&D Center of Hayashibara Co., Ltd. The guidelines for proper implementation of animal studies were adhered.

### Statistical analysis

In human study, blood glucose and serum insulin concentrations used in the statistical analysis were expressed as random minus fasting concentrations. We designated changes in blood glucose and serum insulin concentration to be referred to as Δ(blood glucose) and Δ(serum insulin), respectively. Δ(Blood glucose) and Δ(serum insulin) values were assessed at peak time in ingestion of the carbohydrate solution alone (without IMD). For blood glucose and serum insulin, the incremental areas under the curves (AUC) were calculated for Δ(blood glucose) and Δ(serum insulin) from baseline using the 2 h trapezoid rule. In everted rat intestinal sac test, glucose concentration (0–120 min) and total glucose (120 min) of serosal side were compared between groups without IMD (control group) and with IMD (test group) of KRB buffer solution around sac.

Values of animal study were expressed as mean ± SE, and others were expressed as mean ± SD. In human study, Wilcoxon signed rank tests were employed for statistical analysis. In everted rat intestinal sac test, the time curve of glucose concentrations of serosal side were analyzed using two-way repeated-measures analysis of variance (ANOVA) with treatment and time as within-subjects factors. If time × treatment interactions were significant, data from each point were analyzed using Student’s t-test. Total glucoses of serosal side were analyzed using Student’s t-test. In all tests, the significance level was set at 5%. Statistical analyses were conducted with Statcel2, an add-in software of Microsoft Office Excel.

## Results

### Carbohydrate loading studies

#### Subjects

No subject discontinued from the study because of physical deconditioning related to the study.

#### Effects of IMD in the MD loading study

A study was carried out to examine whether or not IMD would attenuate postprandial blood glucose after ingestion of MD.

##### Blood glucose

**Figure 2.**
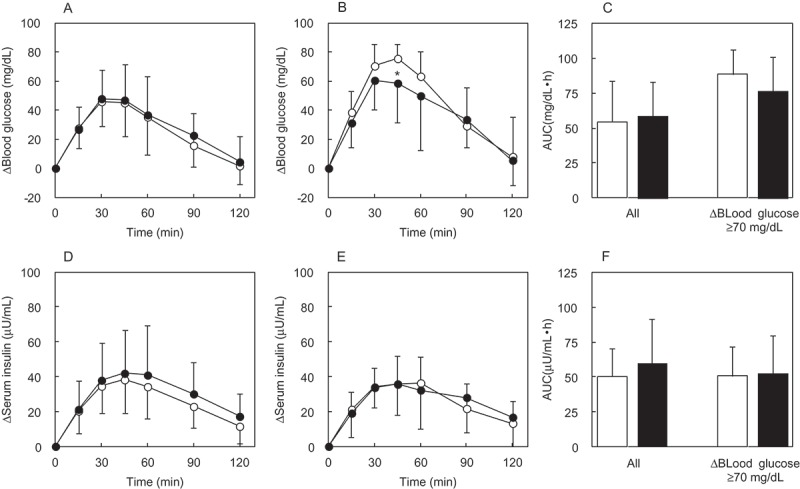
The Δ(blood glucose) and Δ(serum insulin) during the MD loading study. Each subject ingested a solution containing 46.8 g of MD. The time course for the Δ(blood glucose) and Δ(serum insulin) was compared between the MD + IMD (9.6 g) group and the MD alone group. Significant test in the time course of Δ(blood glucose) or Δ(serum insulin) was conducted at a peak time after ingestion of MD alone. Their times were 45 min in Δ(blood glucose), 45 min in Δ(serum insulin) of all subjects, and 60 min in Δ(serum insulin) of subjects with Δ(blood glucose) ≥70 mg/dL. (a) Δ(blood glucose) in all subjects, (b) Δ(blood glucose) in the group of subjects with Δ(blood glucose) ≥70 mg/dL, (c) AUC of Δ(blood glucose) in all subjects and the group of subjects with Δ(blood glucose) ≥70 mg/dL, (d) Δ(serum insulin) in all subjects, (e) Δ(serum insulin) in subjects with Δ(blood glucose) ≥70 mg/dL, (f) AUC of Δ(serum insulin) in all subjects and the group of subjects with Δ(blood glucose) ≥70 mg/dL.○ or white bar: no IMD added, ● or black bar: IMD added. *Significant vs the group without IMD (*p* < 0.05).

The Δ(blood glucose) was reached a postprandial peak at 45 min after ingestion of MD alone (Figure 2(a)). For all subjects, there was no significant difference in Δ(blood glucose) 45 min (a postprandial peak after ingestion of MD alone) after ingestion between the MD + IMD group and the MD alone group (Figure 2(a)). There was no significant difference in blood glucose AUC between the two groups which included all patients. Analysis for the selected subjects in whom the Δ(blood glucose) after MD loading was ≥70 mg/dL (Figure 2(b)), revealed Δ(blood glucose) in the MD alone group reached a postprandial peak at 45 min. For the selected subjects, those in the MD + IMD group at 45 min was significantly (p = 0.04) lower compared with the MD alone group. Blood glucose AUC for the selected MD + IMD group was not significantly different than that in the MD alone group (Figure 2(c)).

##### Serum insulin

The Δ(serum insulin) for all subjects in the MD alone group reached a postprandial peak at 45 min (Figure 2(d)). In the corresponding MD + IMD group, the difference at 45 min was not statistically significant. For all patients, serum insulin AUC was not significant (Figure 2(f)). In the selected subjects with a Δ(blood glucose) ≥70 mg/dL at the time of MD loading, the Δ(serum insulin) was not different between these two groups at 60 min, at which time it was at its maximum for the time course in the MD alone group (Figure 2(e)). Figure 2(f) shows that serum insulin AUC in groups of selected subjects was also not different between groups.

#### Effects of IMD in the sucrose loading study

A study was carried out to examine whether or not the addition of IMD could attenuate blood glucose following sucrose ingestion.

##### Blood glucose

**Figure 3.**
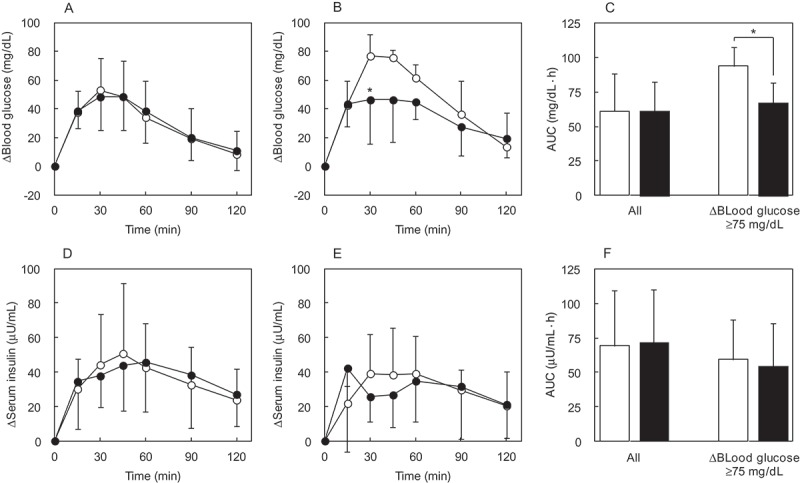
The Δ(blood glucose) and Δ(serum insulin) in the sucrose loading study. Each subject ingested a solution containing 100 g of sucrose. Time course for the Δ(blood glucose) and Δ(serum insulin) was compared between the sucrose + IMD (9.6 g) and sucrose alone group. Significant test in the time course of Δ(blood glucose) or Δ(serum insulin) was conducted at a peak time after ingestion of sucrose alone. Their times were 30 min in Δ(blood glucose), 45 min in Δ(serum insulin) of all subjects, and 30 min in Δ(serum insulin) of subjects with Δ(blood glucose) ≥75 mg/dL. (a) Δ(blood glucose) in all subjects, (b) Δ(blood glucose) in the group of subjects with Δ(blood glucose) ≥75 mg/dL, (c) AUC of Δ(blood glucose) in all subjects and the group of subjects with Δ(blood glucose) ≥75 mg/dL, (d) Δ(serum insulin) in all subjects, (e) Δ(serum insulin) in the groups of subjects with Δ(blood glucose) ≥75 mg/dL, (f) AUC of Δ(serum insulin) in all subjects and the group of subjects with Δ(blood glucose) ≥75 mg/dL. ○ or white bar: no IMD added, ● or black bar: IMD added. *Significant vs the group without IMD (*p* < 0.05).

For all subjects, the Δ(blood glucose) in the sucrose alone and sucrose + IMD group reached a similar postprandial peak at 30 min (Figure 3(a)). Blood glucose AUC was not different between these groups. In the subjects selected based on a threshold glycemic response the Δ(blood glucose) in the sucrose alone group reached a postprandial peak at 30 min as occurred for all subjects administered sucrose alone; however, the magnitude of the Δ(blood glucose) for selected subjects was notably higher at the same time point than it was in the sucrose alone group for all subjects. For the selected subjects, the Δ(blood glucose) of the sucrose + IMD group at 30 min was significantly lower (*p* = 0.03) compared with the sucrose alone group (Figure 3(b)). Blood glucose AUC for selected subjects in the sucrose + IMD group was significantly lower (by about 29%, *p* = 0.02) than that for the selected subjects in the sucrose alone group (Figure 3(c)).

##### Serum insulin

The Δ(serum insulin) in the sucrose alone group, that included all subjects, reached a postprandial peak at 45 min (Figure 3(d)). No postprandial difference in Δ(serum insulin) at 45 min was observed. In the subjects selected for having a peak Δ(blood glucose) ≥75 mg/dL following sucrose loading, and in whom the addition of IMD significantly attenuated postprandial blood glucose, the Δ(serum insulin) was not significant difference (Figure 3(e)). The serum insulin AUC was not different between selected groups of subjects (Figure 3(f)).

#### Effects of IMD on the glucose loading study

**Figure 4.**
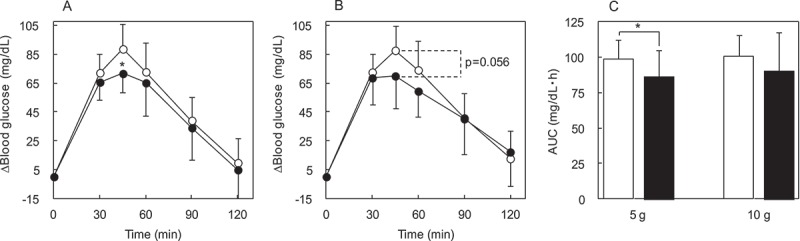
The Δ(blood glucose) following IMD (5 or 10 g) ingestion together with glucose loading. Each subject ingested a solution containing 50 g of glucose. The time course for Δ(blood glucose) was compared between the glucose + IMD (5 or 10 g) group and glucose alone group. Significant test in the time course of Δ(blood glucose) was conducted at a peak time, 45 min after ingestion of glucose alone. (a) Δ(blood glucose) in the glucose + IMD (5 g) group, (b) Δ(blood glucose) in the glucose + IMD (10 g group, (c) AUC of Δ(blood glucose) in the glucose + IMD (5 g and 10 g) groups. ○ or white bar: no IMD added, ● or black bar: IMD added. *Significant vs group without IMD (*p* < 0.05).

To investigate whether IMD affects the absorption of carbohydrate, a glucose loading study was performed. IMD was evaluated for its potential to attenuate postprandial blood glucose during glucose loading. The Δ(blood glucose) in the glucose alone group (*n* = 13–14) reached its postprandial peak at 45 min (Figure 4(a,b)). Compared with glucose alone group, the Δ(blood glucose) in the glucose + 5 g IMD group (n = 13) was significant lower (*p* = 0.02) at 45 min (Figure 4(a)), and the glucose + 10 g IMD group (*n* = 14) tended to be lower (*p* = 0.056) at 45 min (Figure 4(b)). For blood glucose AUC, the addition of IMD made an approximate 13% difference for the 5 g IMD groups and a 10% difference in the 10 g IMD group, and that difference was significant (*p* = 0.046) for the glucose + 5 g IMD group compared with the glucose alone group (Figure 4(c)).

### In vitro IMD digestion test

Digestibility of IMD was 0.0% in human saliva, artificial gastric juice and porcine pancreas. On the other hand, the digestibility of IMD and MD were 42.7% and 86.8% in rat intestinal mucosal enzyme.

#### Inhibition test of digestive enzymes

The inhibitory ratio of IMD for maltase activity were 12.1% (90 mg/dL maltose) and 11.5% (180 mg/dL maltose), respectively. The inhibitory ratio for isomaltase activity were 9.4% (90 mg/dL isomaltose) and 11.0% (180 mg/dL isomaltose), respectively. The inhibitory effect on sucrase was not observed in all tests.

### Glucose absorption test using everted rat intestinal sac

**Figure 5.**
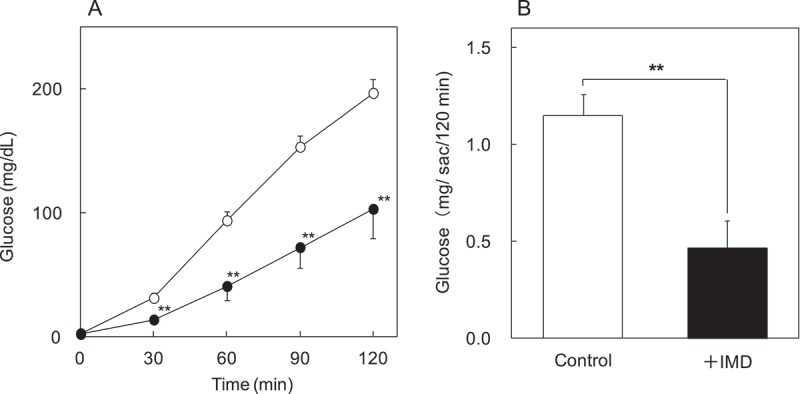
The effects of IMD on glucose absorption *in vitro*. Glucose transport of everted rat intestinal sac was evaluated. Glucose concentration (0–120 min) (a) and total glucose (120 min) (b) of serosal side were compared between groups without IMD (control group) and with IMD (test group) of KRB buffer solution around sac. ○ or white bar: no IMD added, ● or black bar: IMD added. **Significant vs control group (*p* < 0.01).

In the time curve of glucose concentrations of serosal side, time × treatment interactions were significant (*p* < 0.0001). Therefore, data from each point were analyzed between control group and test group. Test group was lower significantly at 30, 60, 90 and 120 min (Figure 5(a)). Total glucose amount of serosal side at 120 min was calculated from glucose concentration and volume of the solution in sac. Total transported glucose of test group (0.47 mg/sac/120 min) was significantly lower (*p* = 0.003) than that of control group (1.2 mg/sac/120 min) (Figure 5(b)).

## Discussion

IMD attenuated postprandial blood glucose in subjects selected for their increased sensitivity to a carbohydrate challenge following MD or sucrose loading. MD is a partially hydrolyzed starch. The staple food contains a lot of starch, and when these are ingested, they are finally digested to glucose and absorbed. It is considered that IMD attenuate postprandial blood glucose by suppressing glucose absorption in the small intestine. Therefore, the ingestion of IMD when added to staple foods such as rice and wheat can be expected to attenuate postprandial blood glucose. Sucrose is one of the most popular sweeteners, and coingestion of IMD with various foods containing sugar (e.g. confectionary) can be expected to attenuate postprandial blood glucose. Furthermore, in the subjects prone to the largest postprandial glucose excursions, the coingestion of IMD (5 or 10 g) with glucose resulted in attenuation of postprandial blood glucose, and the attenuation in coingestion of 5 g IMD was significant. On the other hand, the attenuating effect of 10 g IMD was not a significant difference but a trend (*p* = 0.056). Regarding the relationship between the dose and reaction of physiologically active substances in living organisms, it has been reported that the response becomes a plateau at an excessive dose [17,18]. It is estimated that IMD has a sufficient dose for inhibiting blood glucose absorption in the intestinal tract by about 5 g, and it is expected to be a plateau at higher doses. It has also been reported that blood glucose levels are affected by various factors of daily life in human study [19,20]. Because of these factors, the attenuation of blood glucose level when ingesting 10 g IMD was considered to be a tendency rather than a significant difference. Therefore, we thought that 5 g IMD was enough to attenuate postprandial blood glucose.

Whilst glucose + IMD attenuated blood glucose, RMD did not attenuate postprandial blood glucose [21]. A key mechanism for the attenuation of postprandial blood glucose by RMD is considered to be related to slower glucose absorption through inhibition of the pathway for glucose transport linked to the disaccharidase located in the intestinal epithelium [15,21]. For this reason, it is considered that RMD does not attenuate blood glucose elevations during glucose ingestion. Therefore, the mechanism for the attenuation of postprandial blood glucose by IMD seems to be different from that proposed for RMD.

**Figure 6.**
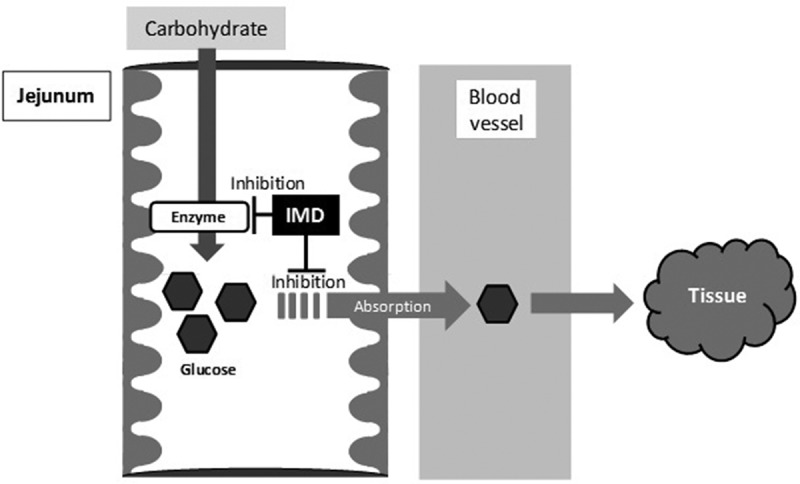
The mechanism of attenuation of postprandial blood glucose in humans consuming IMD. IMD might reduce the magnitude of a postprandial blood glucose excursion after carbohydrate ingestion caused by inhibition of glucose absorption and enzyme activity.

By the way, there are many materials other than RMD to attenuate postprandial blood glucose. By comparing to those of the mechanism, we would like to consider the mechanism that IMD attenuate postprandial blood glucose. First, it was reported that pectin decrease carbohydrate absorption through reducing the affinity between the substrate and small intestine carrier by high viscosity of itself and attenuate postprandial blood glucose [22]. However, it is hard to consider that the viscosity of IMD affects the attenuation of postprandial blood glucose, because the viscosity of IMD is very low (1.4 mPa s: 10% (w/w), 37°C) compared to that of pectin. Next, it was reported that guava polyphenol, black bean extract, and l-arabinose attenuated postprandial blood glucose due to inhibition of carbohydrate digestive enzymes activity in small intestine [23–25]. In this study, IMD slightly inhibited the activity of maltase and isomaltase (about 10%), and did not inhibit the activity of sucrase. Therefore, IMD may slightly contribute to attenuation of the blood glucose excursions by inhibiting these enzyme activities. In this study using everted rat intestinal sac, IMD significantly reduced the absorption of glucose. Therefore, it was suggested that IMD has some influence on inhibition of glucose absorption. Also, it was thought that the influence was the mechanism of postprandial blood glucose attenuation of IMD (Figure 6).

However, if inhibition of glucose absorption after carbohydrate digestion is the only mechanism underlying the attenuating effect of IMD on postprandial blood glucose, the serum insulin of the subjects that have consumed IMD should be lower than in those who have not ingested IMD. In the present study, the subjects in whom IMD attenuated the postprandial Δ(blood glucose) after carbohydrate ingestion had a lower Δ(serum insulin) after sucrose was coingested with IMD, but after MD ingestion Δ(serum insulin) differed little between the groups that did and did not receive IMD. Therefore, it was difficult to consider that the attenuating effect of IMD on postprandial blood glucose is solely attributable to inhibition of glucose absorption after carbohydrate digestion.

## Conclusions

IMD attenuated postprandial blood glucose, not only after the ingestion of MD or sucrose, but also after the ingestion of glucose. As one of the mechanism, it was suggested that IMD inhibited the absorption of glucose on small intestine. These results indicate that IMD can attenuate glucose excursions following ingestion of various carbohydrates at a dose of 5 g. It is thought that the foods and drinks incorporated IMD can contribute to a person worried about blood glucose level.
