# Attenuation of postprandial blood glucose in humans consuming isomaltodextrin: carbohydrate loading studies

**DOI:** 10.29219/fnr.v69.12729

**Published:** 2025-05-20

**Authors:** Tsuyoshi Sadakiyo, Yuki Ishida, Shin-Ichiro Inoue, Yoshifumi Taniguchi, Takeo Sakurai, Ryodai Takagaki, Mayumi Kurose, Tetsuya Mori, Akiko Yamashita-Yasuda, Hitoshi Mitsuzumi, Michio Kubota, Hikaru Watanabe, Shigeharu Fukuda

**Affiliations:** R&D Center, Hayashibara Co., Ltd., Okayama, Japan

## The coauthor name

‘Akiko Yasuda-Yamashita’ is incorrect. ‘Akiko Yamashita-Yasuda’ is correct.

## The legend of Table 1

^‘a^Is data of subject that peak Δ(blood glucose) showed ≥70 mg/dL in MD loading study or ≥75 mg/dL in sucrose loading study’.

This part is incorrect. The following is correct:

^‘a^ Data of subjects that peak Δ(blood glucose) showed ≥70 mg/dL in MD loading study or ≥75 mg/dL in sucrose loading study’.

## ‘Effects of IMD in the MD loading study” under “Carbohydrate loading studies’ of Results section

**Figure:**
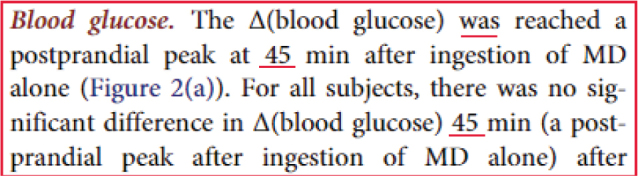


Underlined ‘was’ should be deleted. Underlined number is incorrect. ‘30’ is correct.

**Figure:**
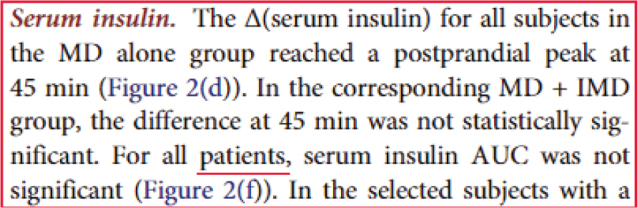


Underlined part is incorrect. ‘subjects’ is correct.

## The legend of Figure 2

**Figure:**
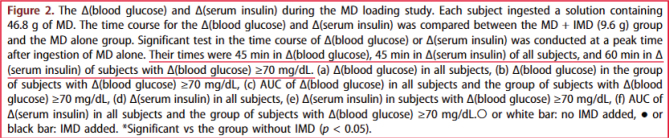


Underlined part is incorrect. The following is correct:

‘Their times were 30 min in Δ(blood glucose) of all subjects, 45 min in Δ(blood glucose) of subjects with Δ(blood glucose) ≥70 mg/dL, 45 min in Δ(serum insulin) of all subjects, and 60 min in Δ(serum insulin) of subjects with Δ(blood glucose) ≥70 mg/dL’.

## ‘Effects of IMD in the sucrose loading study’ under ‘Carbohydrate loading studies’ of Results section

**Figure:**
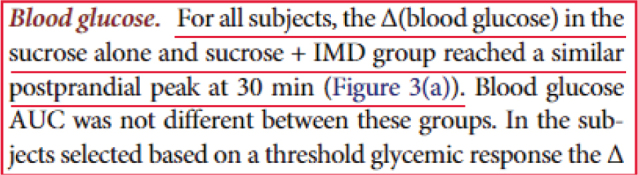


Underlined part is incorrect. The following is correct:

‘The Δ(blood glucose) reached a postprandial peak at 30 min after ingestion of sucrose alone (Figure 3(a)). For all subjects, there was no significant difference in Δ(blood glucose) 30 min (a postprandial peak after ingestion of sucrose alone) after ingestion between the sucrose + IMD group and the sucrose alone group (Figure 3(a))’.

**Figure:**
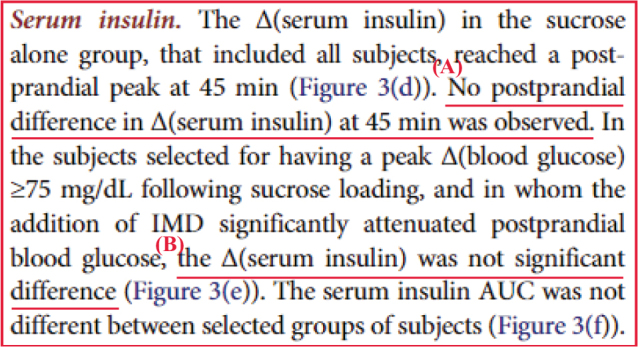


The following sentence is put after sentence (A):

‘For all subjects, serum insulin AUC was not significant (Figure 3(f))’.

Underlined part (B) is incorrect. The following is correct:

‘the Δ(serum insulin) was not significantly different between these two groups at 30 min, at which time it was at its maximum for the time course in the sucrose alone group’

## Figures 2–5

In terms of uppercase or lowercase letter, the alphabet showing each figure does not match one in the legend. Lowercase letter is correct as the alphabet showing each figure.

These errors do not affect the results or conclusions of this article. The authors apologize for any confusion these errors may have caused.

